# SARS‐CoV‐2 Omicron subvariants from BA.2 to BA.2.86 and JN.1: strong lung infection ability and evolving immune escape capacity

**DOI:** 10.1002/mco2.578

**Published:** 2024-06-15

**Authors:** Bixia Hong, Maochen Li, Huahao Fan

**Affiliations:** ^1^ College of Life Science and Technology Beijing University of Chemical Technology Beijing China; ^2^ School of Life Sciences Tianjin University Tianjin China; ^3^ Key Laboratory of Zoonose Prevention and Control at Universities of Inner Mongolia Autonomous Region Medical College Inner Mongolia Minzu University Tongliao China

1

Recently, two back‐to‐back *Cell* papers^1^, ^2^ and two studies published in *Nature* ^3^ and *The Lancet Infectious Disease* ^4^ revealed that the SARS‐CoV‐2 Omicron BA.2 subvariant BA.2.86 exhibited high lung cell tropism and distinct antigenic epitopes, and its sublineage JN.1 even outperformed BA.2.86 in immune escape ability. These findings indicate that the BA.2.86 lineages could pose a potential threat to cause pandemic in the future, which necessitates close monitoring of its evolutionary dynamics.

BA.2.86, a lineage derived from the Omicron BA.2 variant identified on July 24, 2023, is one of the current SARS‐CoV‐2 variants of interest announced by the World Health Organization. JN.1 (BA.2.86+L455S), a sublineage of BA.2.86, has rapidly spread worldwide since its emergence on August 25, 2023, reaching a global prevalence of 72.89% by January 21, 2024 (https://gisaid.org/hcov19‐variants/). Remarkably, compared with the spike protein of BA.2 and XBB.1.5 variants, BA.2.86 sequences from Denmark and Israel have 34 and 36 amino acid mutations (Figure 1A), respectively (https://data.who.int/dashboards/covid19/variants?n=c). And a comprehensive assessment of the biological properties of the BA.2.86 lineage variants is urgently required.

**FIGURE 1:**
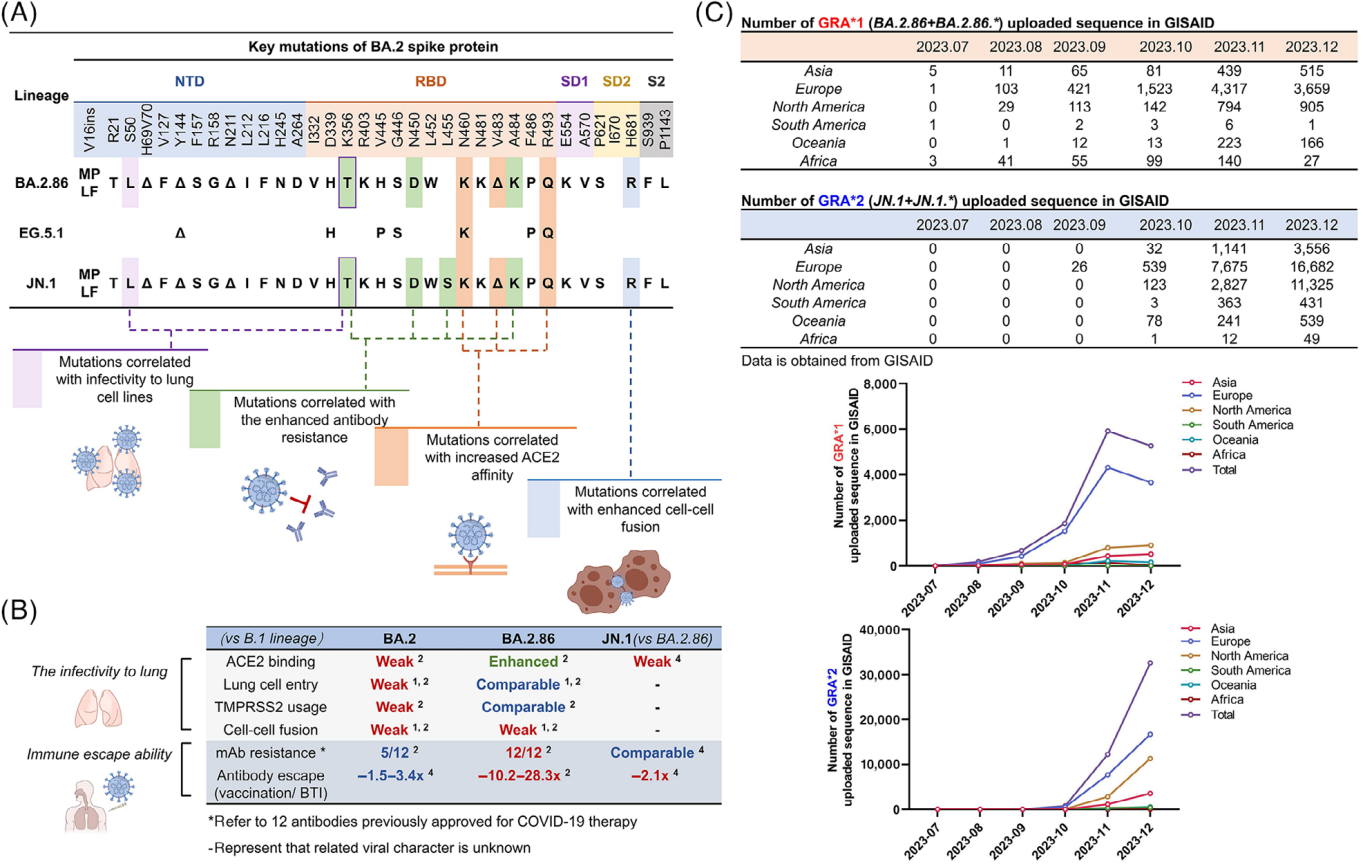
A schematic illustration of the viral characteristics of recent SARS‐CoV‐2 variant BA.2.86 and JN.1. (A) The mutations of emerging variants BA.2.86/EG.5.1/JN.1 are summarized. They are related with the infectivity to lung cell lines (purple), enhanced antibody resistance (green), increased ACE2 affinity (orange), and enhanced cell‐cell fusion (blue). (B) The evolving viral characteristics of BA.2, BA.2.86, and JN.1 in lung cell infectivity and immune escape ability are displayed. The mentioned 12 mAbs are as below: casirivimab, imdevimab, bamlanivimab, etesevimab, cilgavimab, tixagevimab, amubarvimab, romlusevimab, adintrevimab, regdanvimab, bebtelovimab, and sotrovimab. (C) The monthly number of GRA*1 (BA.2.86+BA.2.86.*) and GRA*2 (JN.1+JN.1.*) sequence in GISAID are shown. The emerging cases of GRA*1 and GRA*2 arrived the peak in November and December in 2023, respectively, and the total GRA*2 cases are 3.5 times more than GRA*1, indicating JN.1 and related lineages need to be paid more attentions.

In contrast to other Omicron variants, BA.2.86 exhibits an increased propensity to infect lung cells (Figure 1B). The entry efficiency of the BA.2.86 pseudovirus in 293T‐ACE2 (angiotensin converting enzyme 2) cells was inferior to that of the BA.2 pseudovirus and recently emerged XBB variants (specifically XBB.1.5, EG.5.1, and Flip) pseudoviruses,^1^ and comparable to that of the BA.2 and EG.5.1 pseudoviruses in the Vero with or without ACE2+TMPRSS2 overexpression, and Huh‐7 cells.^2^ Surprisingly, BA.2.86 pseudovirus can enter lung adenocarcinoma cells Calu‐3 and colorectal adenocarcinoma cells Caco‐2 more efficiently than BA.2 pseudovirus and above‐mentioned XBB variants pseudoviruses.^1^, ^2^ Accordingly, BA.2.86 spike protein exhibits higher membrane fusion capacity in Calu‐3 cells than BA.2 spike protein. When effector cells (293T cells with spike plasmid of interest + GFP) were cocultured with 293T‐ACE2 cells, the fusion capacity of BA.2.86 spike protein was not as good as^1^ XBB.1.5 and EG.5.1 spike protein and comparable to^1^ or superior to^2^ BA.2/BA.1 spike protein. Nevertheless, in Calu‐3 cells, the spike protein of BA.2.86 displayed a higher fusion capacity than XBB.1.5 and BA.2/BA.1 spike protein when cocultured with the same effector cells.^1^ Authentic virus infection experiments further confirmed that BA.2.86 was capable of efficiently infecting lung cells (specifically Calu‐3 cells), although with low specific infectivity (the ratio of infectious virus number to virus genome copy number)^2^: Higher levels of genome equivalents were detected in the supernatant of BA.2.86 authentic virus infection than that of B.1 authentic virus infection at 24 h p.i. (virus genome equivalents: BA.2.86 > B.1 > EG.5.1.5 > BA.1). Notably, no viable plaque‐forming unit was detected in the supernatant of BA.2.86 authentic virus infection at 24 h p.i. and 48 h p.i., and only minimal levels were observed at 72 h p.i.. The robust entry of BA.2.86 to lung cells might be also related with its enhanced binding ability to ACE2. Zhang et al.^2^ demonstrated that the spike protein of EG.5.1 and BA.2.86 could bind to soluble recombinant ACE2 more efficiently compared with B.1 and BA.2. Wang et al.^3^ also found that the affinity between ACE2 and the spike protein of BA.2.86 was stronger than that of BA.2, XBB.1.5, and EG.5.1 based on SPR experiments. The robust entry of the BA.2.86 variant may be related to its V445H and R493Q mutations, which introduce hydrogen bonds between the spike protein of BA.2.86 and ACE2.^1^ In addition, S50L (NTD) and K356T (RBD) mutations are considered as the significant determinants of the enhanced lung cell entry of BA.2.86.^2^ The P681R^2^ and A570V^1^ mutations may enhance cell‐to‐cell fusion capacity of BA.2.86 by increasing spike trimer stability.

Notably, JN.1 and BA.2.86 with unique antigenic epitopes exhibit potent immune escape capacity against most monoclonal antibodies available in the market. Even for S309, a monoclonal antibody could neutralize almost all Omicron subtypes, failed to effectively neutralize BA.2.86 pseudovirus.^1^, ^2^, ^3^ Wang et al.^3^ thoroughly analyzed the immune escape capacity of BA.2.86 pseudovirus on monoclonal antibodies against various antigenic epitope and found that BA.2.86 exhibited complete or partial resistance to neutralization of NTD, SD1, and RBD 2 and 3 class epitopes. BA.2.86 exhibited a considerably higher sensitivity to the majority of mAbs against class 1 and class 4/1 epitopes than EG.5.1, among which monoclonal antibodies SA55 and 10−40 against class 4/1 epitopes could efficiently neutralize BA.2.86 pseudovirus.^3^ JN.1 further enhanced the immune escape capacity while retaining the specific antigenic epitopes of BA.2.86, and the L455S mutation in the JN.1 variant enhanced its capacity to evade mAbs against class 1 epitopes.^4^ JN.1 could evade across‐RBD 1, 2, and 3 class antibody with high efficiency.^4^ And only monoclonal antibody SA55 against RBD 4/1 class epitopes still exhibited effective neutralizing activity against JN.1 pseudovirus.^4^ The resistance of BA.2.86 to different classes of antibodies results from a complex interplay of multiple mutations. Specifically, the H245N mutation mediates resistance to neutralizing antibodies targeting the NTD^3^; the E554K mutation facilitates evasion of SD1 class antibodies^3^; the N460K and F486P mutations compromise the neutralizing activity of certain RBD class 1 and/or class 2 monoclonal antibodies^3^; mutations D339H, K356T, V445H, N450D, L452W, and A484K^1^, ^3^ are thought to mediates resistance to some monoclonal antibodies against RBD class 3. However, S50L, I332V, R403K, and R493Q mutations sensitize the BA.2.86 to neutralization by certain mAbs (especially RBD class 1 and class 4/1 mAbs).^3^

BA.2.86 exceeded BA.2 in the immune evasion capacity, but was inferior to XBB.1.5, EG.5.1, and Flip.^1^, ^2^, ^3^ However, the sera of the XBB.1.5‐vaccinated cohort (<1 month) could effectively neutralize BA.2.86 and EG.5.1 pseudovirus, although their neutralization titers were still lower than those against B.1.^2^ Notably, the plasma from XBB infection rehabilitees had 1.1–2.1‐fold lower neutralization activity against JN.1 pseudovirus than BA.2.86 pseudovirus,^4^ indicating the evolving immune escape ability of JN.1. Despite BA.2.86 exhibits a limited immune escape ability to the plasma from XBB.1.5‐vaccinees and XBB infection rehabilitees, the evolving immune escape ability of the BA.2.86 lineages including JN.1 and other variants should be alerted.

In conclusion, these studies investigated some important biological properties of SARS‐CoV‐2 BA.2.86 lineage variants, namely, the stronger lung infection ability and evolving immune escape capacity. Its higher propensity to infect lung cells and ACE2 affinity indicates that the evolutionary trend of SARS‐CoV‐2 does not prefer “lower virulence for better transmission” but shifts to suitable for their own survival. Due to the high rates of antigenic evolution of BA.2.86, this lineage may have the capacity to continuously evolve to the variants that can completely escape the immune response induced by infection or vaccination, and this may be the most adverse case for SARS‐CoV‐2 evolution.^5^ Currently, JN.1 variant with stronger lung infection ability is spreading globally at a fast pace (Figure 1C), it exhibits enhanced immune evasion potential compared with BA.2.86, thus the evolution of JN.1 should be continuously monitored.

## AUTHOR CONTRIBUTIONS

H. F. designed the research. H. F., B. H., and M. L. read the papers and analyzed the data. B.H. and H. F. wrote and revised the manuscript. M.L. drew the figure. All authors have read and approved the final manuscript.

## CONFLICT OF INTEREST STATEMENT

All the authors declare no conflict of interest.

2

## ETHICS STATEMENT

Not applicable.

## Data Availability

Not applicable.
